# Thermoplastic Starch (TPS) Films Added with Mucilage from *Opuntia Ficus Indica*: Mechanical, Microstructural and Thermal Characterization

**DOI:** 10.3390/ma13041000

**Published:** 2020-02-23

**Authors:** Fabrizio Scognamiglio, Daniele Mirabile Gattia, Graziella Roselli, Franca Persia, Ugo De Angelis, Carlo Santulli

**Affiliations:** 1 Technologies and Diagnostics for Conservation and Restoration Laboratory, School of Science and Technology, University of Camerino, Via Pacifici Mazzoni 2, 63100 Ascoli Piceno, Italy; fabrizio.scognamiglio@unicam.it; 2 Department of Sustainability SSPT-ENEA—Casaccia Research Center, Via Anguillarese 301, 00123 Rome, Italy; daniele.mirabile@enea.it (D.M.G.); franca.persia@enea.it (F.P.); ugo.deangelis@enea.it (U.D.A.); 3 School of Science and Technology, Chemistry Division, University of Camerino, Via S. Agostino 1, 62032 Camerino, Italy; graziella.roselli@unicam.it; 4 School of Science and Technology, Geology Division, University of Camerino, via Gentile III da Varano, 62032 Camerino, Italy

**Keywords:** cactus mucilage, thermoplastic starch, bioplastics, extraction process

## Abstract

Opuntia cladodes are a typical vegetable waste, from which mucilage in gel form can be extracted. This work proposes blending it with a self-produced thermoplastic starch (TPS), originating from potato starch with a high content in glycerol (ca. 30%). Three methods were compared for extraction, bare maceration (MA), mechanical blending (ME) and mechanical blending following maceration (MPM) to produce films with an approximate thickness of 150 μm. For the comparison, tensile testing, differential scanning calorimetry and scanning electron microscopy were used. The MPM process proved the most effective, not only for extraction yielding, but also to obtain a larger deformation of the samples with respect to the one allowed by the pure TPS films. A considerable plasticization effect was observed. Despite this, the mechanical performance is still not completely satisfactory, and the expected effect of the calcium and magnesium salts contained in the mucilage to improve the rigidity of the TPS film was not really revealed. Prospected improvements would concern the fabrication process and the investigation of other possible loading modes and sample geometries.

## 1. Introduction

*Opuntia ficus indica*, also referred to as “nopal”, is a plant used in many countries e.g., around the Mediterranean Sea, but also in colder countries, both for ornamental value and for the production of Indian figs, which amount in Italy, according to data supplied from the National Institute of Statistics (ISTAT), to around 750,000–900,000 quintals per year. In most cases, be it for fruits production or for ornamental purposes, it is essential that Opuntia plants are pruned during the winter with the removal of cladodes, i.e., flattened leaf-like stems, which include a mucilage rich also in inorganic components, such as calcium carbonate compounds, and therefore of wide industrial interest for their bioavailability [1]. A number of uses have been suggested for mucilage, a highly branched heteropolysaccharide, for example, as an industrial hydrocolloid as a source of cellulosic fibers, which could have a dietary value when fresh [2]. Another possible use of the mucilage is an industrial hydrocolloid, in which case the extraction method followed presents some importance on its rheological properties [3]. In particular, as far as rheology is concerned, the viscous response appears to be predominant at low frequencies, while the elastic one dominates at high frequencies [4]. In recent years, the food packaging industry has become widely interested in cactus mucilage, proposing its application by developing films and coatings with it [5]. The advantage of using this mucilage, as close as possible to its natural state, only removing the spines and the most fibrous parts, would be its perfect adaptability to contain food, up to the point that even edible films based upon it have been proposed [6]. Plasticizers are normally applied for the purpose of film fabrication, the most diffuse of which is glycerol, normally affecting neither food preservation nor the edibility characteristics, though slightly reducing the films’ rigidity [7].

However, in practical terms, one of the most diffuse applications for cactus mucilage is its use in a blend with another biopolymer of industrial origin. In previous studies, one of the most suitable candidates appeared to be poly(vynilalcohol) (PVA), which was able to yield, in combination with corn starch and glycerol, nopal mucilage films stable up to above 150 °C [8]. Trying to optimize the respective contents of chitosan, PVA and mucilage in the films to improve their resistance, the introduction of more chitosan proved beneficial for the scope [9]. Despite these limitations, using the sole Opuntia mucilage with plasticizers to try to obtain a structural biopolymer film appears to be of interest, in particular, as regards the idea to use as much as possible waste material of biological origin, such as that from the cladode. Attempts have been performed of this way of proceeding, referred to as the production of “Do-It-Yourself (DIY) bioplastics”, mainly based on the introduction of different types of waste. This proved adapted to some uses, such as packaging, biomedical or other structural applications, such as coating, despite the limitation of being hardly able to be used above the gelling temperature of starch e.g., around 80 °C [10,11,12]. In physical terms, these materials are based on starch and glycerol, therefore behaving as thermoplastic starches (TPS) [13]. The idea is that the mucilage could add as an additional plasticizer, if extracted in the most suitable way to improve the plasticization process.

This work proceeds from an extensive study over Opuntia cladode waste, with the idea of globally resulting into a full use of the material obtained. In particular, the crystalline fraction of the cellulose content was disposed of by obtaining nanocellulose fibers [14], while a second study concerned the use of dry nopal fibers to reinforce a starch–glycerol thermoplastic polymer [15]. Here, the examination is completed by evaluating the mechanical and thermal characteristics of Opuntia mucilage extracted with different methods to be introduced again in a TPS matrix. The idea is comparing different modes of extraction of Opuntia mucilage in terms of mechanical performance. It is suggested that modifying a uniform material such a TPS containing a constant value of structural matter (amylose) with the introduction of as-received waste filler would always result in a plasticization effect, to an extent, which is dependent on the mucilage extraction method adopted.

## 2. Materials and Methods

### 2.1. Mucilage Extraction and Preliminary Measurements

Cladodes were received from garden waste collectors, as soon as they were cut from the plant for seasonal pruning and mucilage extraction was carried out using the only parenchyma, the use of the fibrous components present in the surface was already treated in work published in [15].

**Figure 1:**
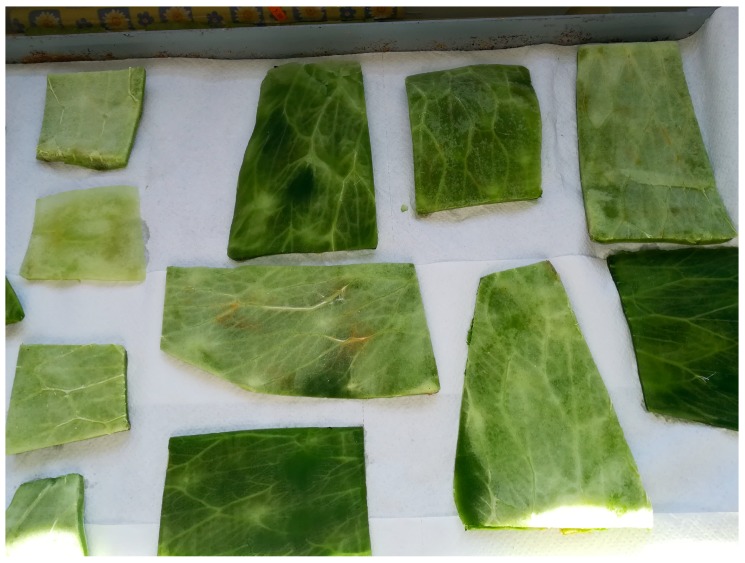
Cladodes after cutting for mucilage isolation, before starting the different procedures.

**Figure 2:**
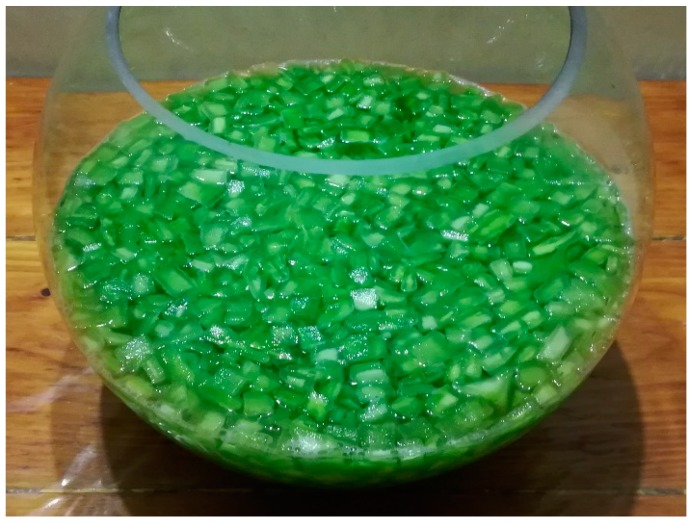
Pieces of isolated parenchyma in a distilled water solution for maceration.

**Figure 3:**
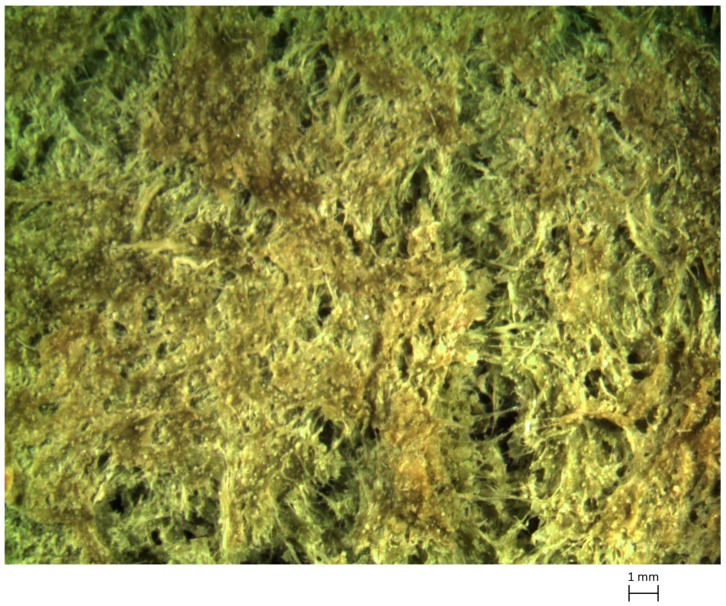
Material retained after 250 μm mesh filtering.

The procedures adopted to extract the mucilage, from the raw material obtained from the cladode, shown in Figure 1, are three:

Maceration (MA): isolation and cutting of parenchyma at ambient temperature in approximately cubic pieces of around 1 cm side (Figure 2), and maceration in distilled water (1 g per mL of parenchymal material) for 24 h in the dark, and separation of the macerated material by different filtration stages, from 2 mm to 250 μm mesh. The macerated material is depicted in Figure 3, after filtration.

Mechanical (ME): the cubic pieces of isolated parenchyma are mechanically treated in an immersion blender for extraction.

Mechanical post maceration (MPM): extraction was performed using a Bosch Msm87110 immersion blender (Bosch, Gerlingen, Germany) using the macerated material separated by filtration. This would allow using the parenchymal material as much as possible. 

In all cases, the pH of the extracted mucilage was in the region of 4.7 ± 0.3, without significant changes. As expected, its behavior was that of a non-Newtonian fluid, and the viscosity of the solution was measured, yielding results in the region of 10 cps for MA extraction, 150 cps for MPM extraction and 220 cps for ME extraction.

Following extraction, the concentration of the mucilage has been carried out in an oven (De Longhi, Treviso, Italy) at 70–75 °C until the complete evaporation of water is achieved, which takes several hours, followed by cooling down at ambient temperature. In this way, different concentrations are obtained: in particular for MA extraction, the concentration is 8 ± 0.5 g of dry mucilage/L, for MPM extraction is 21 ± 1.5 g/L, and for ME extraction is 12 ± 3 g/L. This suggests that in any of the three cases, the concentration of the mucilage led to the removal also of water linked into further components of mucilage, such as carbohydrates, because the amount of free water in the cladode is between 88% and 95% [16]. Despite this, the most conservative approach appears to be MPM extraction.

To summarize, in view of the production of the films, mechanical extraction gives more concentrated mucilage solutions, yet makes more difficult the processing in view of their higher viscosity. No mucilage-only control samples were prepared, for the technical difficulty to process it in the way exposed. It is also noticed that the study would refer to the introduction of garden waste as filler in TPS, and therefore the use of pure mucilage would have also been out-of-focus.

### 2.2. Films’ Preparation

A number of films were prepared:

- A thermoplastic starch (TPS) film was prepared using potato starch, previously extracted from yellow skin cull potatoes with around 25% dry matter, measured at 105 °C, by centrifuging them at 3000 rpm for 15 min, as per indications coming from [17]. The obtained starch contained 23 wt.% amylose and bulk amylopectin, and with an average granule dimension of 50 μm. Starch was filtered before gelatinization under a 250 μm mesh, as to remove moisture-generated lumps. To fabricate the TPS, 10 g of sieved potato starch was added with 4 mL of glycerol, reagent grade, were added, in 400 mL of distilled water. The solution was stirred manually at a temperature around 90 °C to produce gelatinization for approximately 10 min, until its appearance is uniform. It was then subsequently poured in an uncovered steel mold with the approximate surface of 200 × 150 mm covered in Teflon, to ease demolding, and then kept there for an hour at 70 °C, and finally at 45 °C overnight.
- Nopal films with 10 g of sieved potato starch and 4 mL of glycerol, reagent grade, in 400 mL of nopal mucilage, which therefore replaces distilled water, using the same procedure for production, as before. Three different nopal films were produced using the different extraction procedures, therefore defined as maceration (MA), mechanical (ME) and mechanical post-maceration (MPM) films, respectively.

Optical microscopy of the mucilage was carried out, after drying it completely at 75 °C in an oven.

### 2.3. Films’ Characterization

**Figure 4:**
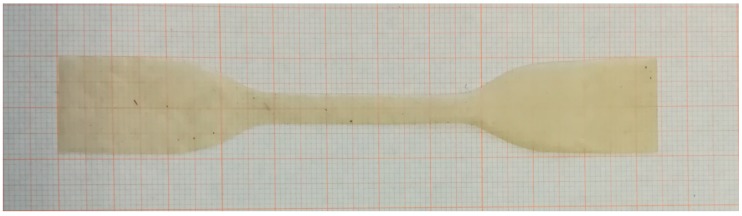
Typical dog bone dimensions (in the image a barely macerated (MA) film sample is shown as an example).

The measurements carried out involved, apart from basic morphological characterization, such as film thickness measurement, other sounder methods of analysis, which included in particular:

- Optical microscopy and scanning electron microscopy (SEM) of the films as received, to evaluate their morphology and after tensile tests, to study the fracture surfaces. The optical microscope used was a NIKON Eclipse 80-i C1 (Nikon, Minato, Tokyo, Japan). The SEM apparatus used was a Zeiss EVO MA15 (Carl Zeiss, Oberkochen, Germany). Using SEM, also a qualitative analysis of the elements present on the surface was carried out by EDS (energy dispersive X-ray spectrometry).
- Tensile tests were performed using the Testometric model MICRO 350 (Testometric Co. Ltd., Rochdale OL11 1NR, UK) displacement control mode at a cross-head velocity equal to 2 mm/min, using dog-bone specimens with a gauge length equal to 40 mm. Typical dimensions of the samples are shown in Figure 4.
- Differential scanning calorimetry (DSC) to evaluate the evolution of the films’ behavior with temperature by heating at a rate of 10 °C/min, from 25 to 180 °C, using the Mettler Toledo HPDSC system (Mettler Toledo, Columbus, Ohio, US). Temperatures for the different transition moments (i.e., onset, peak and ending temperature) were determined using the first derivative of the heat capacity calculated from DSC.

## 3. Results

**Figure 5:**
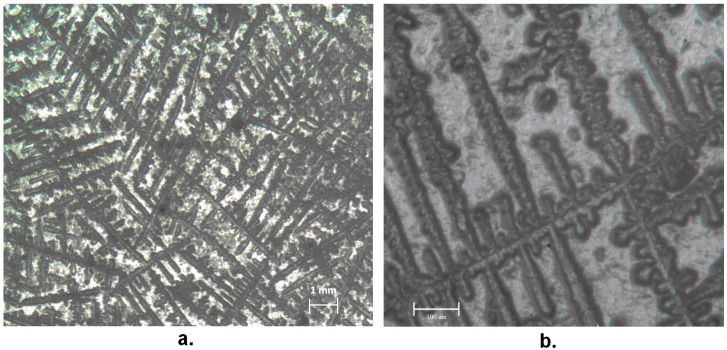
Optical micrographs of dry Opuntia mucilage at different magnifications.

In general terms, the use of cladode material, which is worn out and therefore with a high level of maturity, would imply the presence in it of high levels of calcium salts, as suggested e.g., in [18]. In practice, the possible use of nopal mucilage dispersion to produce edible gels, which requires sufficient strength, despite its viscoelastic behavior, does depend on the combined presence of gel-promoting cations, such as Ca^2+^, with sucrose, resulting in calcium-rich polysaccharides, generally defined as pectins [19]. This gives a strong gelling capacity, defined as the presence of “egg-box” junctions [20]. The optical micrographs of the dried mucilage samples did indicate the presence of very regular three-dimensional structures, of dendritic type, as revealed from Figure 5. Previous studies had been reported that the content of calcium not included in ashes in nopal mucilage is close to 10%, mainly represented by calcium oxalates [21].

The comparison between the three films obtained by blending TPS with the same amount of mucilage needs starting from the consideration that the MPM film offers in principle a better yield in terms of extraction of mucilage over the waste, as reported in Section 2.1. Thickness values are reported in Table 1, together with film compositions obtained by weighing the final samples with an accuracy of ±0.1 g. The data show that after extraction, mucilage filler after extraction is in an amount quite close of the global starch content in the material. It is noteworthy that the MA films obtained, besides being in general terms slightly thicker, does also present very thinner ends, which were removed from the plates and excluded from thickness calculation, and in practice, not used for tensile testing and other observations.

**Figure 6:**
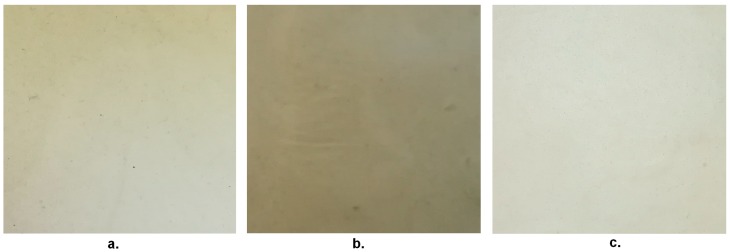
Surface appearance of the three TPS–mucilage films: Macerated (MA) (**a.**), Mechanically extracted (ME) (**b.**), Mechanically extracted post-maceration (MPM) (**c.**).

**Figure 7:**
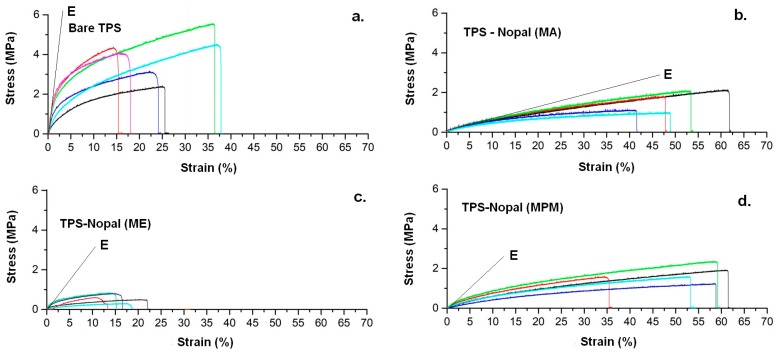
Tensile curves of thermoplastic starch (TPS) (**a.**) and its blended films with nopal mucilage (MA film (**b.**), ME film (**c.**), MPM film (**d.**)). Colors represent the curves for the different samples tested.

After obtaining the films, macrographs of surfaces of differently produced mucilage–TPS blend films were taken. The respective colors would suggest that the one which achieved the least effective homogenizing was the MA film, which retained also more markedly, the original color of the waste used, whereas the best one is the MPM one (Figure 6). Tensile data indicate a large variability between the samples, which could be attributed though to the use of TPS, since the latter does show it as well, as from Figure 7. The absolute data of tensile strength obtained for TPS is higher than the one deemed acceptable by conventional standards for packaging films i.e., 3.5 MPa [22]. However, this is no longer the case for TPS added with mucilage, which on the other side provides a plasticization effect, which may result in the film being more adaptable during its application.

The values of Young’s modulus and of toughness, the latter as obtained from the area under the stress–strain curve, are also reported, although it needs to be observed that a real elastic behavior is only observed, especially in films containing mucilage, only in the very early stage of loading. Therefore, the Young’s modulus approximate average values are obtained by the slope of the intercept with the curves in the initial part of loading, where they appear more or less superposed to each other. As a general consideration, by the introduction of mucilage, the calculated stiffness drops by more than an order of magnitude and elastic behavior is almost immediately lost, more so in the MA films. In terms of toughness, it is worth noting that the MPM film is able to yield values comparable to the pure TPS, although tolerating a much larger deformation of the material.

**Figure 8:**
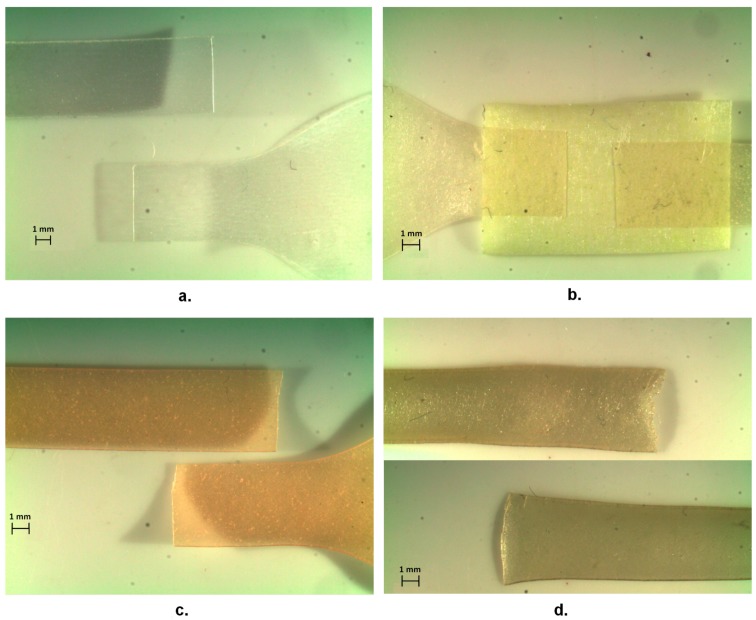
Tensile fractured samples of pure TPS (**a.**) and its blended films with nopal mucilage (type MA (**b.**), type MPM (**c.**) and type ME (**d.**))

**Figure 9:**
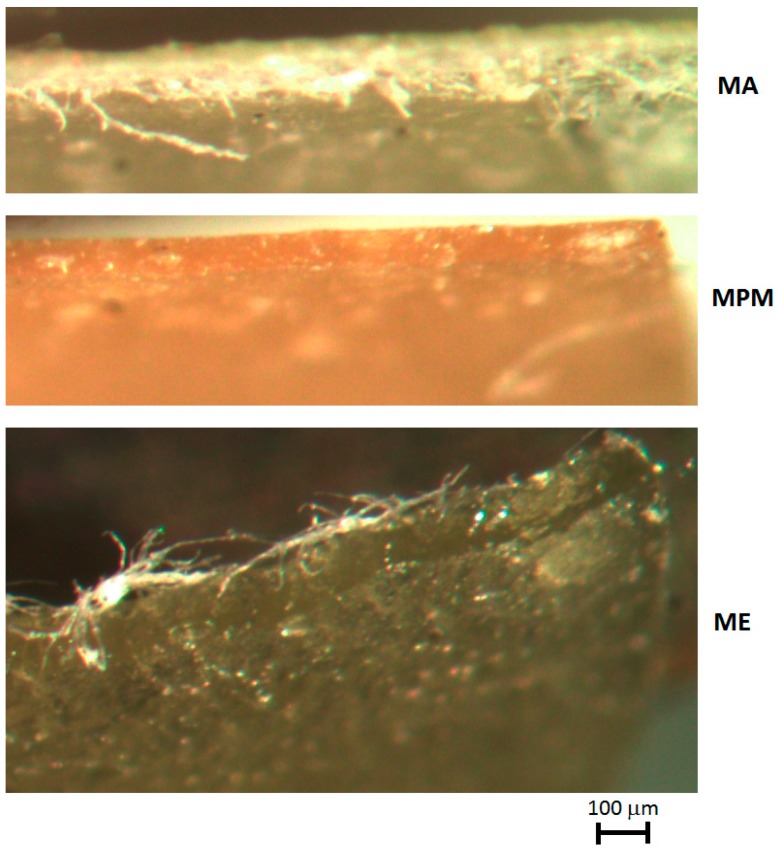
Micrographs of the edges of fractured MA, MPM and ME films after tensile tests.

On the other side, it is evident that the structure of the mucilage does allow obtaining a substantial increase of final elongation, when more thoroughly mixed, therefore, for MA and MPM films (Table 2). Examining the morphology of tensile failure, as from Figure 8, it appears that TPS is evidently brittle, with a straight line of fracture, whereas in the cases of films with nopal mucilage, the lines are always somehow curved, therefore it can be suggested that the filler has some toughening effect. The three different composite films including nopal mucilage were also compared by microscopically observing their edges after tensile fracture (Figure 9). This enabled to carry out the evaluation of the effectiveness of the different extraction procedures on the performance of the final composites. In particular, only the MPM film did not indicate any sign of fibrillation i.e., separation of fibrils around the boundaries of the elementary fibers, during fracture, typical of polysaccharide-based fibrous materials, such as vegetable fibers [23]. The most critical situation was then observed for the ME film, which appeared to be split over the section, during most probably to the presence of air introduced as the consequence of the mechanical extraction of the mucilage, performed starting directly after cutting the pieces of parenchyma into cubes.

**Figure 10:**
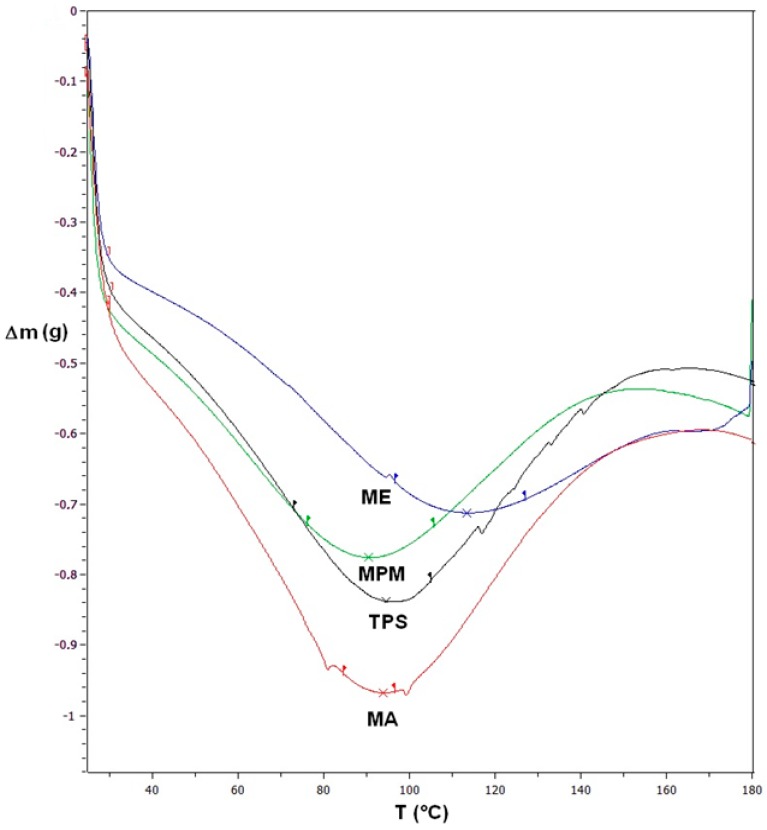
Differential scanning calorimetry (DSC) curves for TPS and its films blended with nopal mucilage (type MA, ME and MPM).

Thermoplastic starches rich in glycerol, like the one used in this investigation, which has a starch/glycerol ratio of 2.5, have characteristically a glass transition temperature below ambient temperature, which means having a thermal transition temperature depending on melting of crystallites [24]. This was determined by DSC (curves are reported in Figure 10 and results are given explicitly with an accuracy of ±0.5 °C in Table 3). It appears that the insertion of nopal mucilage tends in the case of ME films to shift the thermal transition towards higher temperatures, which would in principle extend the usability of the material, though on the other side, its mechanical performance does constitute another limitation. Less significant are the differences obtained in the cases of other blended films, MA and MPM.

The results obtained are comparable with those of other TPS based on potato starch–glycerol system and added with different fillers. In particular, the tensile stress and strain obtained from with MPM films, which can be considered globally the ones in which the introduction of mucilage has the best effect on TPS, is discussed alongside other data available from literature. The reduction of tensile strength by blending TPS with other less-structured polysaccharides, such as mucilage, can be considered typical, in contrast with the reinforcement obtained above a given content of ceramic fillers, such as bentonite [25] or clay [26]. A less pronounced reinforcement effect, although without a complete loss of the plasticization, has been observed also in systems that include microcrystalline cellulose (MCC) as the filler of a potato starch–glycerol (in 100:30 proportion) TPS, using citric acid as a cross-linking agent [27]. This can be attributed to the more uniform distribution of MCC in the film, while this could not be reached with the nopal mucilage, even improving its extraction from the cladode, such as in the MPM process.

**Figure 11:**
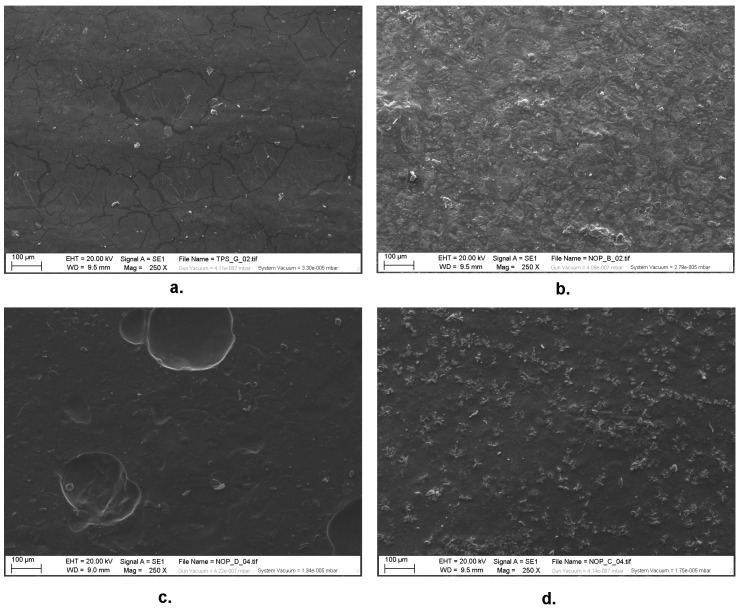
SEM images of TPS film (**a.**) and its blended films (type MA (**b.**), type ME (**c.**) and type MPM (**d.**)). Magnification: 250×.

SEM images of the film surfaces evidenced a lower brittleness of nopal added films with respect to the pure TPS ones, as visible in Figure 11. In contrast, as regards the ME films, the clear presence of air bubbles and the irregularity of the surface does suggest that the use of the blender without previous maceration results also in the large presence of defects, which can explain the poor mechanical performance of these films.

**Figure 12:**
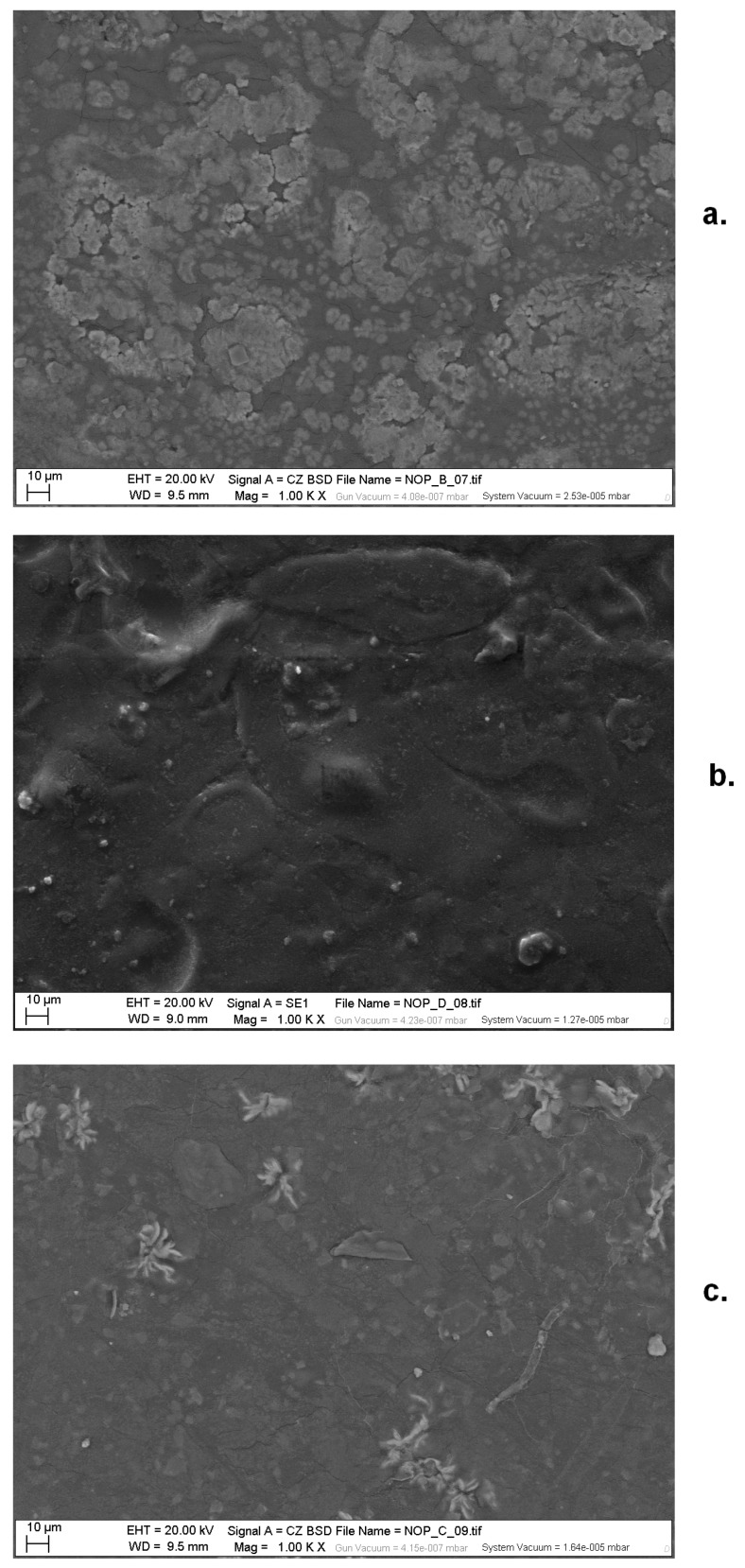
SEM images of blended films TPS–nopal mucilage (type MA (**a.**), ME (**b.**). and MPM (**c.**)). Magnification 1000×.

Despite the aforementioned brittleness, EDS analysis from SEM images highlighted the homogeneity of TPS in terms of retro-diffusion. On the other side, nopal insertion gave evidence of the variety of inorganic salts that are present in this vegetable material. In particular, MA films offered evidence in clear areas of the presence of potassium, sulfur, oxygen, magnesium, aluminum, calcium and chlorine, while in dark areas are present potassium, carbon, oxygen, chlorine, magnesium and traces of aluminum and silicon and sulfur is notably absent. The same elements are present in MPM films, although morphologies appear more visible, in particular flowers, in the clear areas, as reported in Figure 12. Flower structures have been correlated to the presence of calcite in nopal mucilage [28]. In ME films, beyond the same elements as before, also phosphorus, silicon and sulfur are present in the clear areas, but without particularly recognizable morphologies.

## 4. Conclusions

To overcome the limitations of self-produced thermoplastic starches (TPS), based in the specific case of potato starch and glycerol, the addition of nopal mucilage, obtained from Opuntia cladodes waste, has been attempted. Three different ways to extract the mucilage have been explored, maceration, mechanical blending only, and blending after maceration. The latter provided both the highest yielding in terms of mucilage extracted and offered an elongation considerably higher than it is the case for pure TPS: this suggests than an effective plasticization effect was obtained. Moreover, the films obtained with mechanical blending of the mucilage after maceration, defined as MPM, have also toughness comparable to pure TPS, yet allowing considerably higher deformations. In contrast, pure mechanical extraction gave rise to the large presence of defects and air bubbles, leading to ineffective tensile loading. Limits observed are the fact that the production of film blends was not sufficient to improve neither the mechanical strength of TPS nor its variable performance. 

Possible enhancements could be suggested, for example, by employing an acid crosslinker, such as acetic or citric acid, or the mixing with some ceramic particles e.g., talc. Of course the relative amounts have to be optimized, which may require a quite lengthy process, but will be the objective of future work. It might also be suggested that the considerable presence of calcium carbonate and other calcium or magnesium salts would be suitable for the application of other types of loading, such as shear, which could be the objective of further investigation in the future.

## Figures and Tables

**Table 1:** Thickness and composition of thermoplastic starch (TPS) and TPS–nopal mucilage films.

Material	Thickness (mm)	Starch (%)	Glycerol (%)	Mucilage Filler (%)
TPS	0.125 ± 0.015	71.5	28.5	-
MA FILM	0.150 ± 0.009	44.4	11.1	44.5
ME FILM	0.145 ± 0.01	45.6	11.4	43
MPM FILM	0.139 ± 0.008	43.3	10.8	45.9

**Table 2:** Tensile tests results for pure TPS and its blended films with nopal mucilage (type MA, MPM and ME).

Material	Max. Stress (MPa)	Max. Strain (%)	Young’s Modulus (MPa)	Toughness (kNm^2^)
TPS	3.75 ± 1.16	23.4 ± 8.3	240	440 ± 125
MA	1.45 ± 0.48	50.2 ± 7.8	6.5	365 ± 72
ME	0.68 ± 0.22	17 ± 3.5	29	143 ± 41
MPM	1.64 ± 0.36	53.7 ± 10.5	21	442 ± 130

**Table 3:** Thermal transition temperatures of TPS and its films blended with nopal mucilage (type MA, ME and MPM).

Material	Onset Temperature (°C)	Peak Temperature (°C)	Ending Temperature (°C)
TPS	72	95	106
MA	84	93	97
ME	96	112	126
MPM	77	91	108
